# Thrombotic events in COVID-19 patients: A case series and literature review

**DOI:** 10.22088/cjim.11.0.551

**Published:** 2020

**Authors:** Zahra Javidarabshahi, Shohre Khatami, Roxana Rezazade, Neda Saeedian, Mahnaz Mozdourian

**Affiliations:** 1 Lung Diseases Research Center, Mashhad University of Medical Science, Mashhad, Iran; 2 Department of Internal Medicine, Mashhad University of Medical Science, Mashhad, Iran

**Keywords:** Coronavirus Disease 2019, Thrombosis, Myocardial Infarction, Pulmonary Thromboembolism, Venous Thrombosis.

## Abstract

**Background:**

Coronavirus disease 2019 (COVID-19) has rapidly emerged as a threatening pandemic. Clinical features of this pandemic involve a wide range of manifestations and are not completely known. Here, we present six cases of thrombotic events in patients with COVID-19.

**Case presentation:**

In this case series, we present six patients with confirmed COVID-19, according to real-time polymerase chain reaction, who were referred to our emergency department and were found to have thrombotic events. Pulmonary thromboembolism was diagnosed in three patients by computed tomography (CT) angiography. One patient was found to have deep vein thrombosis in Doppler ultrasonography. Another patient who presented with loss of consciousness was finally diagnosed with a cerebrovascular accident using brain CT. One of the patients had developed a myocardial infarction evident on the electrocardiogram.

**Conclusion:**

It is ostensible that COVID-19 puts the patients at an increased risk for developing thrombotic conditions, possibly through formation of hyper-inflammatory and hyper-coagulative states. However, further prospective studies are recommended to confirm these findings.

In December 2019, a novel coronavirus emerged in Wuhan City, Hubei province, China (1). The disease was named as coronavirus disease 2019 (COVID-19), recognized as a global health emergency and subsequently a pandemic by the World Health Organization (WHO) (2). COVID-19 infection can present with a variety of conditions from an asymptomatic disease to a severe illness requiring intensive care unit (ICU) care (3). Although the main presentation of COVID-19 is believed to be respiratory involvement, it should be considered a systemic disease that can involve several organs. Severe COVID-19 infection usually results in a single organ failure, but some patients may develop multiple organ failure (4). A significant underlying condition that has been reportedly associated with COVID-19 is the elevated risk of thrombotic events (5). Patients with COVID-19 infection may develop a hypercoagulable state due to the release of manifold cytokines and the inflammatory state in critically ill patients (6). Elevated hypercoagulability markers, such as the increased level of D-dimer as well as prolonged prothrombin time (PT) and partial thromboplastin time (PTT) were reported in patients with COVID-19 (5, 7). Here, we present six cases of thrombotic events in COVID-19 patients.

## Case Presentation

Herein, we describe six cases of COVID-19 who presented to our emergency department (ED) and were found to have thrombotic events during clinical assessments. Table 1 summarizes the data pertaining to the study cases, including history, clinical characteristic, laboratory and radiological findings, COVID-19 RNA detection result, and the thrombotic events.

**Table 1:** Summary of study cases, including history, clinical characteristic, laboratory and radiologic findings, COVID-19 RNA detection result, and the thrombotic event

	**Case1**	**Case2**	**Case3**	**Case4**	**Case5**	**Case6**	**Reference range**
**Basic information**							
Age (years)	70	38	74	59	38	56	
Sex	Female	Male	Male	Male	Male	Male	
**History**							
Prior thrombotic event	-	-	-	-	-	-	
Comorbid condition	DM	-	COPD	DM	-	-	
**Clinical characteristic**							
Dyspnea	+	+	+	+	+	+	
Cough	-	+	-	+	+	+	
Fever	-	-	-	-	-	+	
Nausea vomiting	-	-	-	-	-	-	
**Laboratory finding**							
WBC(per ml)	9	10	8.2	5.9	5.5	7.4	4.1-10.1
Lymph %	17.4	14	19.8	13	21	19.4	25-45
Neut %	80	81.5	72.2	82	78	72	45-75
Hb (mg/dl)	10.9	16.5	12.3	14.2	14.7	14.3	12-16
Platelet(per ml)	288	143	250	176	149	365	150-400
Urea (mg/dL)	34	36	64	24	23	28	15-43
Creatinine (mg/dL)	0.8	1.3	1.6	1.1	1	1.3	0.3-1.3
LDH )IU/L(	946	411	2406	921	821	986	≤480
AST (U/L)	58	44	49	54	26	58	≤40
ALT (U/L)	35	65	52	35	33	113	≤40
CRP (mg/dL)	193	67	33	121	164	217	≤5
ESR (mm/h)	42	104	34	57	60	64	Male ≤15 Female≤20
O_2 _Sat %	82	86	84	80	83	84	≥94
PH	7.41	7.38	7.31	7.47	7.41	7.4	7.35-7.45
PaO2 (mmHg)	45	36	31	28	19.7	42	80-110
PCO2 (mmHg)	36	40	48	45	37	42	35-45
HCO3 (mEq/L)	23	24	24	33	23	26	21-28
D-dimer (mcg/mL)	700	11075	800	500	950	750	≤500
TPI (ng/ml)	12	20	35866	30	78	6	0-0.4
**Radiologic findings**							
Lung CT	Bilateral GGO	Bilateral crazy paving	Bilateral GGO	Bilateral GGO	OP pattern	Bilateral GGO	
**Detection of COVID-19**							
RT-PCR for COVID-19	+	+	+	+	+	+	
**Thrombotic event**							
	DVT right femoral and popliteal	PTE of right main and all segments	Extensive anterior MI	Ischemic CVA in left basal gang and thalamus	Bilateral PTE in main arteries	PTE in Right Main pulmonary and all segments arteries	

DM: diabetes mellitus, COPD: chronic obstructive pulmonary disease, WBC: white blood cell, Lymph: lymphocyte, Neut: neutrophil, Hb: hemoglobin, LDH: lactate dehydrogenase, AST: aspartate aminotransferase, ALT: alanine aminotransferase, CRP: C- reactive protein, ESR: erythrocyte sedimentation rate, O_2 _Sat: oxygen saturation, TPI: troponin I, RT-PCR: real-time polymerase chain reaction, GGO: ground glass opacification, OP: organizing pneumonia, DVT: deep vein thrombosis, PTE: pulmonary thromboembolism, MI: myocardial infarction, CVA: cerebrovascular accident

**Figure 1:**
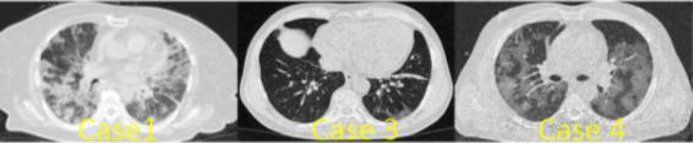
Bilateral ground-glass opacification in lung CT in cases 1, 3, and 4

**Case 1: **The case was a 70-year-old woman who referred to the ED with the sole complaint of dyspnea. The patient did not mention any history of thrombotic events or significant diseases, except for diabetes. Laboratory assessments showed a white blood cell (WBC) count of 9000 per ml with 17.4% lymphocytes and C-reactive protein (CRP) of 193 mg/L. The patient underwent lung computed tomography (CT) that revealed bilateral ground glass opacities (GGO) (figure 1).

Reverse transcriptase polymerase chain reaction (RT-PCR) of the nasopharyngeal swab samples yielded a positive result for COVID-19. On physical examination during the hospitalization period, the patient showed asymmetry in size of her forelegs’ circumference and thus underwent lower limb sonography. Doppler ultrasound exam was consistent with deep vein thrombosis (DVT) in the right femoral and popliteal veins. Subsequent D-dimer and troponin I (TPI) measurements revealed high levels of 700 ng/mL and 12 ng/mL respectively, which confirmed the diagnosis of DVT.

The patient underwent anticoagulation treatment for the diagnosed DVT. Heparin infusion was immediately commenced at a dose of 5000 U, followed by a maintenance dose of 1000 U/h for the first four days. Then, the patient was put on rivaroxaban 15 mg every 12 hours for 3 weeks, which was reduced to 20 mg daily afterwards. The symptoms was resolved completely after three months of follow-up and the patient was in good condition.

**Figure 2:**
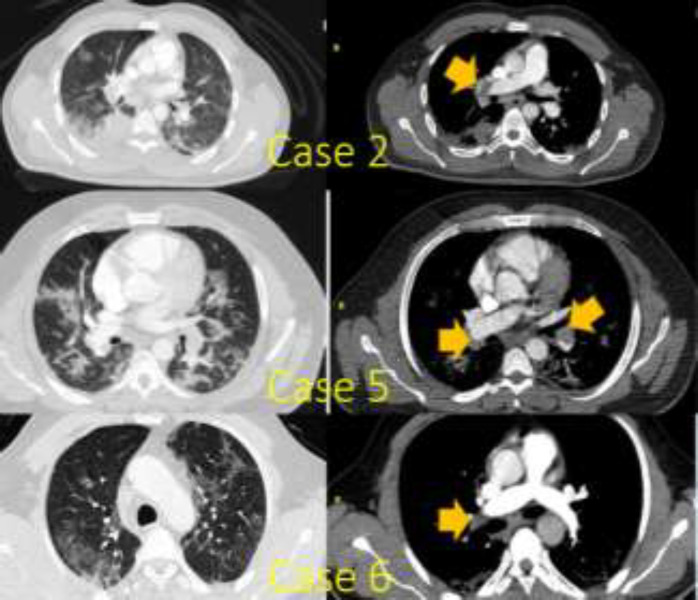
Lung CT scan and CT angiography findings in cases 2, 5, and 6

**Case 2: **Our second case was a 38-year-old man without any significant medical history who presented to the ED complaining of dyspnea and cough. With a clinical suspicion of pulmonary thromboembolism (PTE), the patient underwent CT angiography of the lungs, which showed right pulmonary artery thromboembolism along with bilateral crazy paving pattern in the lung parenchyma (Figure 2). 

Patient’s D-dimer level was 11075 ng/mL and TPI was reported to be 20 ng/mL. We also took a nasal swab sample and performed PCR for COVID-19, which turned out to be positive. Other laboratory assessments revealed a WBC count of 10000 per ml with 14% lymphocytes and an elevated CRP level of 67 mg/L. The patient received anticoagulant therapy with warfarin (5 mg daily for 5 days per week and 7.5 mg on the other two week days) for his PTE and was discharged in good condition with no further complications in a three-month follow-up.

**Case 3: **The third case was a 74-year-old man with a history of chronic obstructive pulmonary disease (COPD) who presented with chest pain and dyspnea. Upon admission, a blood sample was taken from him and tested for TPI, which revealed a level of 35866 ng/mL. His electrocardiography (ECG) showed an extensive anterior myocardial infarction (MI). The patient was admitted with an impression of MI. Blood analysis during hospital stay indicated a WBC count of 8200 per ml with 19.8% lymphocytes and a CRP level of 33 mg/L. As the patient had a persistent dyspnea with low O_2_ saturations, he underwent lung CT scan, which showed a bilateral GGO pattern (figure 1). Therefore, nasopharyngeal swab sample for PCR was taken from him that tested positive for COVID-19. During his stay at the hospital, the patient developed ventricular tachycardia and unfortunately died in spite of undergoing cardiopulmonary resuscitation and receiving electrical cardioversion.

**Figure 3:**
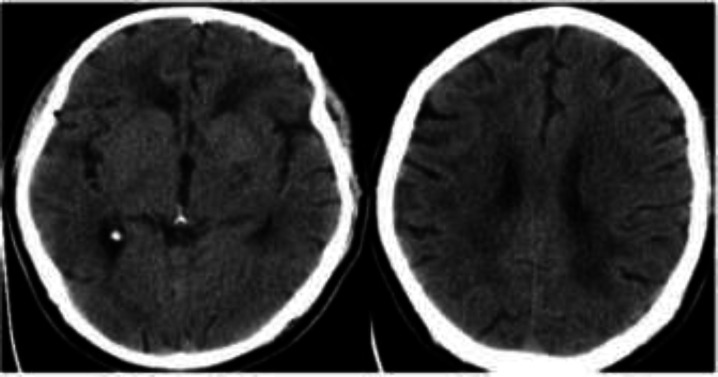
Ischemic cerebrovascular accident in the basal ganglia and thalamus of the left hemisphere in brain CT of case 4

**Case 4: **A 59-year-old diabetic man with a chief complaint of dyspnea and cough showed up at our ED. Initial physical examination revealed an oxygen saturation of 80%. Blood analysis showed a WBC count of 5.9 per ml with 13% lymphocytes and a CRP level of 121 mg/L. Due to his clinical manifestations as well as the high CRP, lymphopenia, and low O_2_ saturations, the patient underwent chest CT with a high clinical suspicion of COVID-19. The CT image showed a bilateral GGO pattern (figure 1). The RT-PCR test of the nasopharyngeal sample was also positive for COVID-19. During his hospitalization period, the patient’s consciousness level dropped markedly. Thus, he underwent a brain CT that showed an ischemic cerebrovascular accident (CVA) in the basal ganglia and thalamus of the left hemisphere (figure 3). As the patient had a late presentation and the golden time for thrombolysis had passed, anti-ischemic treatment was commenced for him with aspirin (80 mg daily), Plavix (75 mg daily), and atorvastatin (40 mg daily). However, the patient’s condition deteriorated and he eventually died in the hospital due to severe acute respiratory distress syndrome (ARDS) and septic shock.

**Case 5: **The case was a 38-year-old man who had dyspnea and cough upon entrance into the ED. His medical history was reportedly uneventful. Complete blood count test showed a WBC count of 5.5 per ml with 21% lymphocytes. Other laboratory investigations showed high serum levels of D-dimer (950 ng/mL) and CRP (164 mg/L). Lung CT images showed an organizing pneumonia pattern and the swab sample from nasopharyngeal mucosa was assessed with RT-PCR and tested positive for COVID-19. General condition of the patient deteriorated during the course of hospitalization and he developed respiratory distress. Therefore, the patient underwent CT angiography of the lungs with a high clinical suspicion of PTE, which confirmed the diagnosis of bilateral PTE in main pulmonary arteries (figure 2). The patient initially received heparin and then he was discharged in good condition with warfarin therapy at a daily dose of 2.5 mg. After three months of follow-up, he had no further complications.

**Case 6: **The case was a 56-year-old male who referred to the ED with fever, dyspnea, and cough. He was severely ill upon admission and thus was immediately transferred to ICU. Laboratory tests showed a WBC count of 7.4 per ml with 19.4% lymphocytes and a CRP level of 217 mg/L. Chest CT scan showed a bilateral GGO pattern (figure 2) and the RT-PCR of the nasal swab was positive for COVID-19. During the course of treatment, the patient’s lymphopenia alleviated, but respiratory distress was still present. A chest CT angiography was carried out for the patient, which was consistent with PTE in the right main pulmonary artery (figure 2). D-dimer level was found to be 750 ng/mL. This patient was also discharged in good condition with a prescription for rivaroxaban (15 mg daily) and showed no complications in a 3-month follow-up.

## Discussion

With the advent of COVID-19, clinicians primarily recognized it as a respiratory infection that often presents as an atypical pneumonia. However, as few months have passed and the body of evidence has grown larger, we have attained a more comprehensive knowledge of COVID-19 and its potential manifestations. The disease may present itself through involvement of different organs, which can lead to single or multiple organ failures (4).

Although several studies have shown thrombocytopenia in COVID-19 patients, it may also present with coagulopathy in some patients (8). On the one hand, venous stasis due to the bedridden condition of COVID-19 patients was primarily suggested to be the underlying etiology of this hypercoagulable state. On the other hand, the first presentation of COVID-19 was reported to be a thrombotic event in some patients (9), as was the case in some of our patients who initially presented with a thrombotic event and later on were diagnosed with COVID-19 during their hospitalization. It seems that the severe inflammatory response along with extensive cytokine release are associated with a hypercoagulable state that causes thrombotic events in these patients (5). Furthermore, hypoxia is another factor that contributes to the exacerbation of the condition in these cases (10). This hypercoagulable state may eventually end in disseminated intravascular coagulation (DIC) in some patients (11). Klok et al. (12) assessed the incidence of thrombotic events in patients with COVID-19 who were admitted to the ICU and reported that despite proper anticoagulant therapy, thrombotic events happened in 31% of the patients (27% with venous origin and 4% in the arteries). They reported that pulmonary embolism was the most frequent complication in these patients. The authors proposed PT > 3 seconds or activated PTT > 5 seconds as the risk factors for thrombotic events in COVID-19 infected patients. These findings are in line with what we observed in our case series, as half of our patients had PTE and some even presented with a PTE as their first clinical manifestation and turned out to be infected with COVID-19 afterwards.

D-dimer has also been suggested as a prognostic marker in patients with COVID-19 infection (5). Some even proposed it as a routine laboratory examination that should be conducted in COVID-19 patients (13). In a large multicenter study in China, high levels of D-dimer was found in 46.4% of COVID-19 patients (43% in non-severe cases and 60% in severe ones) (3). Huang et al. also proposed that COVID-19 patients with D-dimer levels more than 2.4 mg/L are more prone to thrombotic events (13). Nearly all cases in our study had elevated levels of D-dimer, which indicates a possible role for this marker in the pathobiology of the disease.

It is believed that COVID-19 causes massive fibrin formation that may precipitate in different tissues and subsequently produce high levels of D-dimer as the fibrosis resolves. The precipitated fibrin in alveolar spaces and interstitial tissue of the lung along with thromboses in small vessels result in adverse events such as respiratory failure that eventually requires intubation (5, 14).

Zhang et al. (15) reported a 69-year-old man with COVID-19 and a history of hypertension, diabetes, and CVA. The patient primarily had a moderate infection, but his condition gradually deteriorated and eventually led to intubation. CT showed multiple new infarcts in the brain and laboratory tests showed elevated levels of D-dimer and antiphospholipid antibodies. Likewise, we had a case of CVA with high levels of D-dimer. This hypercoagulable and hyperinflammatory state may cause instability in the atherosclerotic plaques in different vessels. Unstable plaques may in turn rupture and cause different cardiovascular events such as MI (16), as was the case in one of our patients.

Davoodi et al. (17) reported a 57-year-old woman who presented with leg swelling and non-frequent dry cough during the few days leading to her presentation. On examination, she had dilated and thrombotic veins in her calf. Ultrasonography of the lower extremities was suggestive for DVT. When the patient underwent lung CT to rule out PTE, patchy opacities appeared in the CT image. She was tested positive for COVID-19 RNA in the RT-PCR of nasal swab sample. This scenario implies that COVID-19 infected patients may initially present with thrombotic events.

In conclusion, it seems that COVID-19 infection predisposes patients to coagulopathies and various thrombotic events. Some patients may even present with these findings as their primary manifestation. These thrombotic events seem to occur regardless of traditional risk factors, as some patients showed no predisposing factor in their history. It seems that the hyperinflammatory and hypercoagulative states are responsible for the presence of thrombotic events. However, this association should be further assessed in prospective studies.
